# Is CaMKII friend or foe for cell apoptosis in eye?: A narrative review

**DOI:** 10.1097/MD.0000000000036136

**Published:** 2023-12-01

**Authors:** Weixing Xu, Hua Liu

**Affiliations:** a School of Graduate, Dalian Medical University, Dalian, China; b The Third Affiliated Hospital of Jinzhou Medical University, Jinzhou, China; c School of Graduate, Jinzhou Medical University, Jinzhou, China.

**Keywords:** apoptosis, CaMKII, excitotoxicity, eye, high glucose, oxidative stress

## Abstract

Ca^2+^/calmodulin-dependent protein kinase II (CaMKII) controls cell proliferation, differentiation, apoptosis, and other biological processes that have an essential role in eye diseases. However, it seems that previous studies have generated conflicting conclusions about the effect of CaMKII on cell apoptosis. In this review, we explore the positive and potentially deleterious effects of CaMKII on eye cell apoptosis. We can safely conclude that the early elevation of CaMKII could be viewed as a promoter of cell apoptosis. Overexpression of CaMKII by transfection or pretreatment with drugs helped combat cell apoptosis.

## 1. Introduction

Calcium ion (Ca^2+^) regulates an array of cellular processes as a highly versatile intracellular signal.^[1,2]^ Ca^2+^/calmodulin-dependent protein kinase II (CaMKII) controls cell proliferation, differentiation, apoptosis, and other biological processes that have an essential role in eye diseases.^[2,3]^ Recently, Bo Chen team^[2]^ published a study in the journal Cell, which concluded that CaMKII is a key regulator of retinal ganglion cell (RGC) survival in normal and diseased retinas, including excitotoxic injury to RGC somas, optic nerve injury to RGC axons, and glaucoma. These results were amazing and made us very interested in the potential use of CaMKII in ophthalmic diseases. However, it seems that previous studies have generated conflicting conclusions about the effect of CaMKII on cell apoptosis.^[4–7]^ For example, CaMKII may induce neuronal apoptosis under some conditions.^[6,7]^ Whereas under other conditions, CaMKII inhibition protects RGCs from excitotoxic cell apoptosis.^[4,5]^ In this review, we explore the positive and potentially deleterious effects of CaMKII on eye cell apoptosis.

## 2. The subtypes and structure of CaMKII

**Figure 1.**
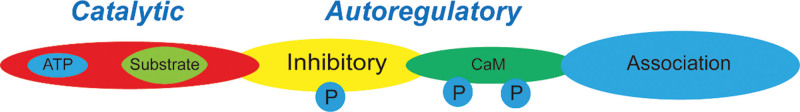
Structural representation of a prototypical CaMKII subunit. CaMKII = Ca2+/calmodulin dependent protein kinase II.

CaMKII has 4 isoforms (α, β, γ, and δ) identified in human tissues.^[8]^ These isoforms are expressed differently in tissues. In eye, α and β are highly expressed in retina nerve cells.^[2,9]^ And γ and δ are mainly expressed in vascular endothelial cells and other cells.^[8,10–15]^ As shown in Figure 1, each isoform of CaMKII consists of a catalytic domain, an inhibitory domain, a Camodulin (CaM) binding domain, and an association domain. And the inhibitory domain and the CaM binding domain are called autoregulatory domains.^[16–18]^ All isoforms had 89% to 93% similar amino acid sequences in their catalytic and autoregulatory domains.^[8]^ The catalytic domain contains adenosine triphosphate (ATP) binding sites and other substrate anchorins. In the inhibitory domain and CaM binding domain, they both have autophosphorylation sites that are in different positions. In the inhibitory domain, the autophosphorylation site of the α isoform was Thr286, and the β, γ, and δ were Thr287. In the CaM binding domain, the autophosphorylation site of the α isoform was Thr305/306, and the β, γ, and δ were Thr306/307. In the resting state, the binding of the inhibitory domain to the catalytic domain will inhibit the release of ATP and make CaMKII inactive because no Ca^2+^ binds to the CaM binding domain.^[16–18]^ When Ca^2+^ binds to the CaM binding domain, the autophosphorylation site of the inhibitory domain is subject to autophosphorylation. And then the catalytic domain releases ATP and activates CaMKII.^[17]^ At this point, CaMKII activity is no longer dependent on Ca^2+^ or CaM; that is, CaMKII remains partially active and phosphorylates adjacent CaMKII when calcium ions are removed.^[19–21]^

## 3. CaMKII and cell apoptosis

Apoptosis, also known as programmed cell death, is involved in the occurrence, development, and extinction of many eye diseases. RGC apoptosis in glaucoma leads to irreversible vision loss. Photoreceptor cell apoptosis occurs after retinal detachment, and its visual function is difficult to recover even after surgical reduction. Retinitis pigmentosa leads to the death of photoreceptor cells due to gene mutations activating the apoptotic mechanism.^[22]^ CaMKII was involved in apoptosis induced by various factors, such as high glucose (HG), oxidative stress, excitotoxicity, and ocular hypertension. CaMKII regulates apoptosis through endoplasmic reticulum protein phosphorylation, Fas receptor activation, and mitochondrial apoptosis.^[23,24]^

### 3.1. Apoptosis induced by high glucose

In macaque choroid-retinal endothelial cells (RF/6A), CaMKII activation was induced by HG and then led to the activation of both Fas-dependent and mitochondria-dependent apoptosis pathways.^[24]^ Leukemia inhibitory factor (LIF) significantly inhibited HG-induced human retinal endothelial cells (HRECs) apoptosis by down-regulating the CaMKII/CREB signal pathway.^[25]^ Autocamtide-2-related inhibitory peptide (AIP) as a specific inhibitor of CaMKII inhibited the apoptosis of pericytes in the diabetic retina.^[26]^ Curcumin attenuated HG-induced retinal neuron apoptosis by downregulating CaMKII.^[27]^ So far, there have been few studies on the role of CaMKII in apoptosis induced by HG (as described in Table 1). And the conclusions were consistent: CaMKII activation promoted apoptosis in the HG environment, and inhibition of CaMKII activity by an inhibitor or drug could reduce apoptosis.

**Table 1:** Effect of CaMKII on apoptosis induced by high glucose.

Authors and PMID	Model (cell/animal)	Means of intervention	Signal pathway	Effect of CaMKII on apoptosis	The eye disease involved
Jun Li et al^[24]^ PMID: 23049237	Macaque choroid-retinal endothelial cells (RF/6A)	KN93(inhibitor of CaMKII)	CaMKII − JNK − Fas	CaMKII activation promoted apoptosis KN93 inhibited CaMKII activity and reduced apoptosis	DR
Lei Wang et al^[25]^ PMID: 32658632	Human retinal endothelial cell (HRECs) and diabetic mice	Leukemia inhibitory factor (LIF), KN93	CaMKII-CREB	CaMKII activation promoted apoptosis LIF and KN93 down-regulate the protein level of p-CaMKII to inhibit apoptosis	DR
Young Hee Kim et al^[26]^ PMID: 21331776	Pericyte in the retina of diabetic mice with early-stage disease	AIP (inhibitor of CaMKII)	Not described	CaMKII activation promoted apoptosis inactivation of CaMKII by AIP contributes to inhibit apoptosis	DR
Jun Li et al^[27]^ PMID: 26207889	Neural cells in the retina of diabetic rats	Curcumin	CaMKII-caspase-3	CaMKII activation promoted apoptosis Curcumin downregulating CaMKII attenuated apoptosis	DR

AIP = Autocamtide-2-related inhibitory peptide, CaMKII = Ca^2+^/calmodulin dependent protein kinase II, DR = diabetic retinopathy, LIF = leukemia inhibitory factor.

### 3.2. Apoptosis induced by oxidative stress

Overproduction of reactive oxygen species is induced by Ultraviolet B (UVB) and blue light. CaMKII inhibitor KN93 blocked cell death, which was mediated by apoptosis-inducing factor (AIF) caused by blue light.^[28]^ In human lens epithelial cells (HLECs), inhibition of cartilage acidic protein 1 (CRTAC1) expression resulted in downregulation of CaMKII expression and eventually reduced apoptosis induced by UVB irradiation.^[29]^ Glutamate up-regulated cell apoptosis through excessive reactive oxygen species generation. In the differentiated Y-79 cells, CaMKII was activated in the glutamate-induced cell injury. Puerarin can dose-dependently attenuate cell apoptosis through the inactivation of CaMKII.^[30]^ In glutamate-treated cells, CaMKIIalpha (B) increases transiently in the early phase. AIP protected RGCs from glutamate-mediated cell death through the inactivation of caspase-3.^[31]^ Interestingly, for RGCs in stressful environments, basal levels of CaMKII can constantly afford them protection.^[32,33]^ In RGCs with knocked-down levels of CaMKIIalphaB by specific siRNA, cell apoptosis was increased by glutamate. Overexpression of CaMKIIαB by pIRES-hyg3/αB enhanced RGC-5 cell survival against glutamate treatment.^[32]^ However, some studies also found that activation of CaMKII can promote cell survival in a stressful environment. Paeoniflorin (PF) up-regulated the level of p-CaMKII and then triggered AMPK activation against apoptosis induced by oxidative stress in human retinal pigment epithelial (ARPE-19) cells.^[34]^ Combined with Table 2, it can be found that in an oxidative stress environment induced by various factors, the effect of CaMKII activation or inhibition on cell apoptosis was not completely consistent. Under the effects of pathological environments such as blue light, UVB, and glutamate, increased CaMKII activation promoted apoptosis. Cell apoptosis was decreased with CaMKII inhibitor KN93, AIP, and drugs, or with inhibition of upstream protein expression. However, overexpression of CaMKII by transfection or pretreatment with the drug reduced cell apoptosis in oxidative stress, which was induced by glutamate and atRAL, although the effects of the 2 on p-CaMKII levels were opposite. Correspondingly, cell survival decreased induced by glutamate under the knockdown of the CaMKII premise. As described previously, the basal levels of CaMKII may afford cells some ongoing protection against stress.^[32,33]^ So, we speculate that in oxidative stress environments, there could be an indication of a cause-and-effect relationship between the early elevation of CaMKII and the later detection of cell apoptosis. We could treat the early elevation of CaMKII as a trigger for apoptosis. As the apoptotic genes turn on, CaMKII levels decrease, coincident with cell apoptosis. This allows us to understand why cellular CaMKII activation is increased in the oxidative stress environment, but the overexpression of CaMKII is resistant to oxidative stress damage.

**Table 2:** Effect of CaMKII on apoptosis induced by oxidative stress.

Authors and PMID	Model (Cell/Animal)	Means of intervention	Signal pathway	Cause and effect relationship	Effect of CaMKII on apoptosis	Isoform	The eye disease involved
Dawei Yang et al^[28]^ PMID: 33940381	R28 cells	KN93; KN93 were added to the medium at the beginning of blue light exposure	CaMKII-Drp1	Blue light and KN93 intervene at the same time; Changes in CaMKII activity are not described.	CaMKII inhibitor KN93 blocked cell apoptosis.	Not described	AMD, glaucoma
Yinghong Ji et al^[29]^ PMID: 27855397	lens epithelial cells (HLECs)	CRTAC1 siRNA; The UVB treatment was executed, and then the CRTAC1 siRNA construct was transfected into HLECs.	CRTAC1-CaMKII	First, UVB→ CaMKII activity was up-regulated; then, CRTAC1 siRNA→CaMKII activity was down-regulated.	Downregulation of CaMKII expression reduced UVB irradiation induced-apoptosis.	Not described	Cataract
Ke Wang et al^[30]^ PMID: 27409614	The human retinoblastoma cell line Y-79	KN93; Cells were pretreated with puerarin for 24 h and then exposed to glutamate for 24 h.	CaMKII/ASK-1/JNK/p38	First, puerarin → p-CaMKII was down-regulated; then, glutamate →p-CaMKII was up-regulated.	CaMKII activation promoted apoptosis; Puerarin Attenuated Glutamate-Induced Activation of CaMKII-Dependent Apoptosis Signaling Pathway.	Not described	Retinal degenerative disease
Wei Fan et al^[31]^ PMID: 16023257	RGC-5 retinal ganglion cell line	glutamate, AIP; AIP was added to the culture medium 1 h prior to the 24 h exposure to various concentrations of glutamate	CaMKII-caspase3	First, AIP→ p-CaMKIIα was down-regulated; then, glutamate→ p-CaMKIIα was up-regulated.	The expression of CaMKIIalpha(B) gene increased in glutamate-treated cells. Treatment with AIP protected RGC from glutamate-mediated cell death.	Alpha isoforms	Retinal degenerative disease
Wei Fan et al^[32]^ PMID: 17652761	RGCs were obtained from the retinas of Sprague-Dawley rats, RGC-5 Cells	pIRES-hyg3/αB; RGC-5 cells were induced to overexpress CaMKIIαB and then the cells were treated with glutamate for 24 h. siRNA; The purified RGCs were transfected with specific siRNA and then the cells were treated with glutamate for 24 h.	CaMKII-BDNF	First, pIRES-hyg3/αB→ CaMKIIalphaB was up-regulated; then, glutamate →CaMKII was up-regulated. First, siRNA → CaMKIIalphaB was down-regulated; then, glutamate →CaMKII was up-regulated.	The expression levels of CaMKIIalphaB became increased in response to glutamate treatment. Overexpression of CaMKIIαB in RGC-5 Cells Enhanced Cell Survival against Glutamate Treatment. RGCs with knocked down levels of CaMKIIalphaB, a glutamate stimulus led to an increase in cell death.	Alpha(B) isoforms	Retinal degenerative disease
Xue Zhu et al^[34]^ PMID: 30117083	ARPE-19	Paeoniflorin (PF); Cells were pretreated with PF for 6 h before exposure to atRAL for 12 h	CaMKII-AMPK	First, PF→ p-CaMKII was up-regulated; then, atRAL→p-CaMKII was down-regulated. First, PF → p-CaMKII was up-regulated; then, KN93 →p-CaMKII was down-regulated.	The PF up-regulated the level of p-CaMKII against apoptosis.	Not described	AMD

AIP = Autocamtide-2-related inhibitory peptide, AMD = age-related macular degeneration, AMPK = AMP-activated protein kinase, atRAL = all-trans-retinal, BDNF = brain-derived neurotrophic factor, CaMKII = Ca^2+^/calmodulin dependent protein kinase II, CRTAC1 = cartilage acidic protein 1, HLECs = human lens epithelial cells, PF = Paeoniflorin, RGC = retinal ganglion cell, UVB = ultraviolet b.

### 3.3. Excitotoxicity induced apoptosis

In the excitotoxicity model induced by N-methyl-D-aspartate (NMDA), activation of CaMKIIα was essential for protecting RGCs.^[2]^ However, previous studies found that CaMKII activity increased in NMDA-induced neuronal apoptosis. AIP attenuated caspase-3 activation and significantly improved retinal ganglion cell survival.^[4,5]^ In addition, the activity of CaMKII is not immutable in NMDA-induced apoptosis (as described in Table 3). After intravitreal injection of NMDA, the activity of CaMKII was elevated for short time periods, such as 30 minutes or 2 hours. But CaMKII activity decreased after 24 hours.^[33]^ Moreover, CaMKII activity changes differently under different concentrations of NMDA. For example, after 2 hours of intravitreal injection of 20 mM NMDA, CaMKII activity is down-regulated,^[2]^ while under 4 mM NMDA, CaMKII activity is up-regulated.^[5]^ It can be seen that the change in CaMKII activity was closely related to the intensity and action time of external adverse factors. There are multiple genes, including apoptosis-associated genes involved in excitotoxicity apoptosis, which can be regulated by AIP.^[35]^ In pathological settings, AIP contributes to inhibiting cell apoptosis. However, in the absence of external adverse factors, the intravitreal injection of AIP resulted in a partial loss of RGC activity.^[2]^ So, it reasonable to think that AIP inhibits cell apoptosis by regulating the expression of apoptosis-associated genes, not only CaMKII. Therefore, we cannot make the conclusion lightly that there were conflicting results about the role of CaMKII in cell survival.

**Table 3:** Effect of CaMKII on apoptosis induced by excitotoxicity.

Authors and PMID	Model (Cell/Animal)	Means of intervention	Signal pathway	Cause and effect relationship	Effect of CaMKII on apoptosis	Isoform	The eye disease involved
Xinzheng Guo et al^[2]^; PMID: 34297923.	C57BL/6 mice RGCs	Intravitreal injection for AAV2-mediated gene transfer of CaMKII α/β was performed at 2 weeks before NMDA (20 mM) injection	CaMKII-CREB	First, AAV-CaMKII α/β→ CaMKII α/β was up-regulated; then, NMDA→p-CaMKII was down-regulated at 2 h after NMDA injection.	Activation of CaMKIIα/β is essential for protecting RGCs; inactivation of CaMKII by AIP contributes to apoptosis of RGCs.	α/β	Neurodegenerative diseases such as glaucoma and diabetic retinopathy
A Laabich et al^[5]^; PMID: 11146104.	Sprague–Dawley female rats RGCs	AIP was administered 2 h before NMDA injections and also coincident with the injection of NMDA (4 mM)	CaMKII-caspase3	First, AIP→ CaMKII-independent activity was down-regulated; then, NMDA→CaMKII-independent activity was up-regulated at 2 h after NMDA injections. AIP and NMDA were injected simultaneously.	CaMKII-alpha(B) isoform plays a role in excitotoxicity-induced neuronal apoptosis; Neuroprotection achieved by AIP.	alpha(B)	Neurodegenerative diseases such as glaucoma and diabetic retinopathy
A Laabich et al^[33]^; PMID: 10762700.	Sprague–Dawley female rats retinal neurons	A single intravitreal injection of NMDA (4 mM)	not described	NMDA→activity of CaMKII and the CaMKII-alpha(B) mRNA levels were up-regulated at 30 min or 2 h after NMDA injections. NMDA→CaMKII activity and in the CaMKII-alpha(B) mRNA levels were down-regulated at 24 h after NMDA injections.	The activity of CaMKII and the CaMKII-alpha(B) mRNA levels were found to be elevated for short time periods (30 min, 2 h) after a single intravitreal injection of NMDA (4 mM). Decrease in CaMKII activity and in the CaMKII-alpha(B) mRNA levels at longer time periods (24 h) following injection of NMDA (4 mM).	alpha and alpha(B)	Neurodegenerative diseases such as glaucoma
Dennis J Goebel^[4]^; PMID: 19135986.	Male Sprague-Dawley rats RGCs	Pretreatment of AIP into the vitreous 2 h prior to NMDA-treatment	CaMKII-caspase3	The expression level of CaMKII was not described after modeling.	AIP fully attenuates caspase-3 activation for at least 8 h following NMDA (20 nmol) insult and also significantly improves retinal ganglion cell survival.	alpha	Neurodegenerative diseases such as glaucoma and diabetic retinopathy

AAV = adeno-associated virus, AIP = Autocamtide-2-related inhibitory peptide, CaMKII = Ca^2+^/calmodulin dependent protein kinase II, NMDA = N-methyl-D-aspartate, RGC = retinal ganglion cell.

### 3.4. Apoptosis due to ocular hypertension

In the chronic intraocular pressure elevation rat model, brain-derived neurotrophic factor (BDNF) can induce beneficial synaptic changes. Expression of CaMKII, as a key molecule involved in synaptic changes, was upregulated after BDNF injection.^[36]^ Further, RGCs degenerate in glaucoma models, which include elevated intraocular pressure or genetic deficiency. And reactivation of CaMKII through intravitreal AAV-CaMKIIα injection protects RGCs.^[2]^ In terms of the above findings (as described in Table 4), activation of CaMKII can help reduce cell apoptosis. Unfortunately, there are few studies on CaMKII in glaucoma at present, and more evidence is needed to support this view.

**Table 4:** Effect of CaMKII on apoptosis induced by ocular hypertension.

Authors and PMID	Model (Cell/Animal)	means of intervention	signal pathway	Effect of CaMKII on apoptosis	The eye disease involved
Hae-Young Lopilly Park et al^[36]^; PMID: 31142572	Sprague-Dawley rats cauterization surgery (three episcleral veins were cauterized using a standard technique)	IOP elevation BDNF (brain-derived neurotrophic factor)	CaMKII-CREB	Expression of CaMKII were upregulated after BDNF injection which induce beneficial synaptic changes in glaucoma	Glaucoma
Xinzheng Guo et al^[2]^; PMID: 34297923	C57BL/6 mice was injected magnetic microbeads into the anterior chamber to occlude aqueous outflow. Mice deficient in the glutamate transporter Glast (GLAST¯/¯), a model of normal tension glaucoma.	IOP elevation/genetic deficiency intravitreal AAV injection	CaMKII-CREB	Reactivation of CaMKII protects RGCs. Inactivation of CaMKII has no improvement of RGC survival.	Glaucoma

AAV = adeno-associated virus, BDNF = brain-derived neurotrophic factor, CaMKII = Ca2+/calmodulin dependent protein kinase II, IOP = intro-ocular pressure, RGC = retinal ganglion cell.

## 4. Conclusions

In summary, current evidence shows that CaMKII plays an important role in cell apoptosis. The change in CaMKII activity was closely related to the stimulus intensity and action time of external adverse factors. The early elevation of CaMKII could be viewed as a promoter of cell apoptosis. Overexpression of CaMKII by transfection or pretreatment with drugs helped combat cell apoptosis.

## Author contributions

**Formal analysis:** Weixing Xu.

**Investigation:** Weixing Xu.

**Methodology:** Weixing Xu.

**Project administration:** Hua Liu.

**Resources:** Hua Liu.

**Supervision:** Hua Liu.

**Writing – original draft:** Weixing Xu.

**Writing – review & editing:** Hua Liu.
